# Acceptance and Commitment Therapy (ACT) intervention in adolescents with type 1 diabetes: A pilot and feasibility study

**DOI:** 10.1177/13591053251328961

**Published:** 2025-03-31

**Authors:** Kirsten R Panton, Keely Bebbington, Amy Finlay-Jones, Tiziana Bufacchi, Maria Davey, Marita Smith, Leanne Fried

**Affiliations:** 1 School of Psychological Science, The University of Western Australia, Australia; 2 The Kids Research Institute Australia, Australia; 3 Centre for Child Health Research, The University of Western Australia; 4 Child and Adolescent Health Service, Perth Children's Hospital, Australia; 5 Curtin University, Australia

**Keywords:** Acceptance and Commitment Therapy, ACT, diabetes, intervention, mindfulness

## Abstract

A considerable proportion of patients with type 1 diabetes (T1D) experience emotional problems due to the continual demands of the disease, which may persist throughout life without appropriate support. The aim of this study was to assess the feasibility and acceptability of an Acceptance and Commitment Therapy (ACT) intervention and provide early indications of its capacity to impact psychosocial outcomes for adolescents with T1D. Twenty-two participants were randomised to one of two groups: (1) ACT intervention for 6 weeks or (2) standard care. Given the ease of recruitment, high rate of attendance for the ACT intervention and the positive responses to the intervention, the intervention was deemed feasible and acceptable to adolescents with T1D. Outcomes trended towards enhanced Quality of Life and reduced Diabetes Distress. Future work should consider a larger scale randomised controlled trial to investigate the efficacy of the intervention for T1D and other chronic illnesses.

## Introduction

In addition to the challenges typically associated with adolescence, youth with type 1 diabetes (T1D) are at risk for poor physical and mental health outcomes as they assume autonomy for managing an intensive treatment regimen, and navigate the transition from paediatric to adult healthcare services (Geist et al., 2003). In the absence of treatment, psychological distress during emerging adulthood may persist throughout life, thereby substantially increasing the long-term personal and social burden of living with T1D (Sawyer et al., 2007). Accordingly, there is a documented need for interventions designed to promote psychological wellbeing and prevent psychological distress that are tailored to the unique needs of adolescents with T1D (Winkley et al., 2020). Poor adherence to diabetes management has been associated with poorer health-related quality of life (HR-QoL; Chantzaras and Yfantopoulos, 2022).

Mindfulness-based interventions may be an effective means for enhancing psychological wellbeing and reducing distress in adolescents with T1D. Mindfulness can facilitate the detection of early signs of stress, ruminative thoughts, and physiological arousal by enhancing self-awareness (Baer, 2003). Interventions that focus on enhancing mindfulness have been shown to improve both emotion-regulation and self-regulation, and are associated with reductions in stress, depression, externalizing behaviours, and sleep problems in adolescents deemed at risk for a difficult transition into adulthood (Bluth et al., 2016; Eva and Thayer, 2017; Shomaker et al., 2017). The impact of mindfulness-based interventions on emotion-regulation and self-regulation is of particular importance to adolescents with T1D. T1D requires self-regulation skills to support adherence to arduous daily treatment regimens, and adolescents often have poor adherence to T1D self-management (Rausch et al., 2012). However, it is essential that adolescents assume increased responsibility for managing the disease as they progress to adulthood.

Acceptance and Commitment Therapy (ACT) is a mindfulness-based intervention that focuses on noticing the ongoing, transitory process of thinking, without acting to alter the content of the thoughts or the emotional response to the thoughts. As many adolescents seek increased autonomy, they may be particularly receptive to the methods of ACT, which explicitly focus on personal responsibility, choice, and values (Hadlandsmyth et al., 2013). ACT also aims to enhance psychological flexibility, which refers to an individual’s ability to respond adaptively to changing situational demands in a manner that maintains the pursuit of a valued goal (Kashdan and Rottenberg, 2010). Psychological flexibility may involve shifts in mindset and the consideration of competing desires or goals (Doorley et al., 2020; Hayes et al., 2004; Kashdan and Rottenberg, 2010). Psychological flexibility could be an important factor in promoting resilience in people with a chronic condition (Gentili et al., 2019). Importantly, for adolescents with T1D, psychological flexibility was found to be associated with glycaemic control, quality of life and depressive symptoms (Alho et al., 2021, 2022).

ACT has been tested as an intervention for chronic physical diseases, such as type 2 diabetes, epilepsy, chronic pain, and obesity, with studies indicating positive results for psychological and physical health outcomes following intervention (Prevedini et al., 2011; Swain et al., 2015). Limited, although promising studies using ACT interventions have been conducted with adolescent populations with T1D (Barreto et al., 2019; Barry-Menkhaus et al., 2020). Recent research has supported the use of brief interventions (single sessions) using ACT-based exercises in improving adolescent health behaviours and T1D self-management (Barreto et al., 2019; Barry-Menkhaus et al., 2020). Additionally, an ACT intervention was found to improve glycaemic control and symptoms of anxiety in adolescents with poorly controlled T1D (Alho et al., 2022). Further studies are needed to strengthen the evidence and better understand the contribution of ACT interventions, which can help young people with T1D navigate the often-difficult adolescent period.

This pilot and feasibility project investigated the use of a face-to-face six-week ACT tailored intervention, in adolescents with T1D. This study aimed to collect feasibility data to inform future clinical trials. This data included intervention recruitment rates, attendance rates, drop-out rates and intervention acceptability. This study was not designed to measure the effectiveness of the intervention, though outcome data will be reported, to provide early indications of the interventions impact on psychosocial outcomes.

## Methods

### Participants

In total, 183 patients of the Perth Children’s Hospital Diabetes Service were contacted via email to participate in the study. Adolescents were eligible if they were an Australian resident aged between 12 and 15 years (inclusive) with a diagnosis of T1D, had an email address and had internet access (via mobile phone, computer, or tablet). Individuals were excluded if they had a reduced cognitive capacity without ability to consent/assent, were involved in concurrent research projects that may impact on the outcomes of this project or were receiving regular assistance from a psychologist or psychiatrist. To obtain precision estimates around the mean and standard deviation to inform an adequately powered future trial, we estimated that a sample size of 10 per group would be suitable for this pilot (Whitehead et al., 2016).

Twenty-two participants were enrolled in the study. The mean age at baseline was 12.81 years (range = 11.9–14 years; SD = 0.31). Five of the participants were male. The mean duration of T1D was 5.9 years (range 1.12–12.33) and mean HbA1c was 7.6% (SD = 0.52). The socioeconomic status, or relative advantage or disadvantage compared to other areas of the suburbs the participants lived in ranged from low (*n* = 2), medium (*n* = 12) to high (*n* = 6), as determined by the Socio Economic Indicators for Areas (SEIFA) (Australian Bureau of Statistics, 2018). Two participants identified English as their second language.

### Ethical considerations

Ethics approval was obtained from the Child and Adolescent Health Service Human Research Ethics Committee. This project followed the principles outlined in the Declaration of Helsinki.

All participants provided informed written consent to participate. Parental consent was obtained for all adolescents involved.

### Intervention

The ACT intervention consisted of six (weekly) ninety-minute face-to-face sessions conducted at the Perth Children’s Hospital in a seminar room with flexible furniture arrangements. The DNA-V protocol (Discoverer, Noticer, Advisor and Values; Rayner et al., 2017) was used for the intervention, which is based on an adaptation of the ACT Hexaflex model (six core components of ACT). Although also used with adults, the DNA-V model was specifically designed for implementation with adolescents. The primary facilitator, who held a PhD in education psychology, attended a three-day training with ACT practitioners prior to delivering the intervention. Each session of the intervention explored an ACT-specific theme designed to promote psychological flexibility and was adapted by the research team to include examples related to living with T1D. A session on self-compassion was also included, given ACT’s consistency with this concept (Neff, 2012) and prior research demonstrating that individuals with chronic illness who report high levels of self-compassion, demonstrate adaptive coping in response to stress (Boggiss et al., 2020; Loseby et al., 2022; Prentice et al., 2021; Sirois et al., 2015). The sessions were: (1) Intro to the DNA-v model and working in groups, (2) the Noticer, (3) the Advisor, (4) self-compassion, (5) values, and (6) the Discoverer and putting the model together (see Table 1 for further details). The protocol was delivered by the primary facilitator, and each session was co-facilitated by a registered psychologist. The psychologist participated in the session but attended in case a participant experienced psychological distress. In this scenario, the psychologist and participant could talk in an adjacent room and the participant would be given the option to return to the group or to withdraw. In either case, the participant’s parents would be notified, and follow-up contact by the psychologist would be undertaken. At the end of each session, participants were thanked for attending and reminded of the psychological support offered through the diabetes team at the Perth Children’s Hospital.

**Table 1.** Outline of goals and activities in the ACT intervention.

Session number and theme	Session goals	Session outline and activities
1. Introduction	•Build community and increase sense of comfort for teens •Cultivate attention and awareness •Understanding of the 3 systems we operate in and what happens when they get out of balance •Learn some strategies to help move out of and into different systems	•Introductions and overview of program •*Activity:* Welcoming name game •Developing ground rules •Introducing Gilbert’s three circles and exploring these •*Activity:* Group discussion around reasons for doing the course
2. The mind	•Participants to understand that the brain is the ultimate survival tool however there are things about the way the mind works that can hijack us •Understand that struggling with thoughts can make them worse •To learn mindfulness as a strategy to help defuse from thoughts	•Our tricky minds: Struggling with thoughts •*Activity:* Watching Caveman mind video •*Activity:* Group discussion – what happens to caveman mind when we have a chronic illness? •*Activity:* The Chinese Fingertrap and Purple Elephant exercises •*Activity:* Soles of the feet mindfulness exercise
3. Defusion and acceptance	•Observe your thoughts rather than hooking into your thoughts – they are merely sounds, words, stories passing through our minds •Thoughts may or may not be true, important or wise – we don’t have to believe them, we only pay attention to them if they are important, and we don’t automatically have to follow their advice •Thoughts are not orders – we don’t have to obey them •Thoughts are like junk e-mail – You can’t stop it from coming in, but you don’t have to read it •Recognising emotions in others and in ourselves •Learning to accept emotions rather than avoiding them and techniques for doing this	•Defusion of thoughts •*Activity:* Group discussion – Defusion of thoughts •*Activity:* Hands as thoughts exercise •*Activity:* Watch internal hijackers video OR Passengers on a bus video •Acceptance of difficult feelings •*Activity:* Mindfulness breathing exercise
4. Self-compassion	•Help participants understand self – compassion through experience of how we treat other •Understand that others hurt in the same way as we do and also just want to be happy like us	•*Activity:* How would I treat a friend? •*Activity:* Common humanity vignettes •*Activity:* Mindfulness – here and now stone exercise
5. Values and behaviours	•Encourage participants to reflect on what is important to them •Have participants start to develop a sense of how values can influence behaviour	•Introducing values •*Activity:* Values card sort •*Activity:* Group discussion – figuring out what natters •Values into action •*Activity:* Values on a bracelet activity
6. Moving forward	•To reinforce what has been learnt over the weeks including the concept of interconnectedness. •To help the participants to find ways to include what they have learnt into their lives •To celebrate their commitment and engagement with the course	•*Activity:* Discussion of what has been learnt of the weeks •*Activity:* Discussion around interconnectedness and the experiences of others •*Activity:* Drama/art activity showing how they may now respond to situations or feelings they may struggle with

### Assessments

*Feasibility* was measured by the length of time taken to recruit the required number of participants, attendance rates, and study drop-out rates. *Acceptability* was assessed through a focus group with participants who received the intervention. Focus group methodology was chosen as a means of eliciting personal accounts and allowing ideas to be developed and articulated through discussion (Kruger and Casey, 2008). They can be particularly appropriate for adolescents who may feel more supported and confident in a group situation (Vaughn et al., 1996). Examples of questions asked in the focus group were: Do you feel you benefited from the group sessions? In what way did you benefit or why didn’t you? What aspects of the intervention did you like or not like? Do you think other people your age with T1D may be interested in attending these sessions?

#### Outcome data

This study was not adequately powered to determine the efficacy of the intervention, however, data on key outcome measures were collected to inform the development of future clinical trials. The psychosocial outcomes measured were quality of life, mindfulness and stress. Attaining good quality of life as well as strict glycaemic control is a challenge for adolescents with T1D (De Wit et al., 2008) and quality of life self-reports provide useful material for assessing emotional and social functioning (Pinquart, 2020). Mindfulness was measured as it shows promise as a potentially protective characteristic against the influence of stressful events on emotional well-being (van Son et al., 2015) and is taught through ACT interventions. Stress, specifically diabetes distress, was measured as it has the potential to negatively impact both the adolescent’s experience of T1D as well as typical developmental tasks (Rechenberg et al., 2017). No statistical analyses were performed on the outcome data.

##### Quality of life

The Paediatric Quality of Life scale (short-form, QoL; Varni et al., 1999) was utilised to assess QoL. The Paediatric QoL scale is a valid (Desai et al., 2014) and reliable measure on a 5-point Likert scale from 0 (never) to 4 (almost always). A sum of the scores from the 15-items was used to create an Overall Score, with higher scores representing higher QoL (Cronbach’s α = 0.88).

##### Mindfulness

The Child and Adolescent Mindfulness Measure (CAMM; Greco et al., 2011) measured mindfulness, using a 5-point scale ranging from 0 (never true) to 4 (always true). The CAMM is considered to be a reliable (Cronbach’s α = 0.81) and valid mindfulness measure in adolescents (Kuby et al., 2015). A total score was calculated from the 10-items, with higher scores indicating a higher level of mindfulness skills.

##### Stress

The Diabetes Distress Scale (DDS; Polonsky et al., 2005) is a 17-item measure, that asks participants to rate their distress on a 6-point Likert scale, from 1 (no problem) to 6 (serious problem). Higher scores represent greater distress (Cronbach’s α = 0.93). The DDS has been validated for use in various languages (Chew et al., 2015; Chin et al., 2017; Farm et al., 2017), and has demonstrated good internal reliability (Polonsky et al., 2005).

### Procedure

Twenty-two participants consented and were randomised into either the Treatment Group (*TG*; ACT intervention) or Control Group (*CG*). Two participants (one from each group) had to withdraw before baseline data collection as they elected to be involved in another study. *TG* (*n* = 10) received the face-to-face ACT intervention for 6 weeks and standard care, while *CG* (*n* = 10) received standard care. Three participants from the *CG* withdrew before the six-week data collection point; one for family reasons and two did not want to be in the control arm of the study. At 6 weeks, the *CG* (*n* = 7) received a subscription for a Mindfulness App while the *TG* (*n* = 10) received standard care during this time. There was no *CG* from 6 to 12 weeks. Data were collected at baseline *(CG and TG)*, six-weeks *(CG and TG)* and 12 weeks (*TG only)* through an online survey sent to participants, with two reminders for completion.

A focus group was conducted with *TG* participants on completion of the six-week face-to-face ACT intervention. The focus group, conducted by an experienced facilitator, was audio-recorded and transcribed verbatim.

### Data analysis

Dependability and credibility were maintained by ensuring the focus group transcript was checked for accuracy (by author LF and an independent researcher),and analysed with the assistance of the qualitative research software package QSR NVivo, Version 12 (QSR International, Burlington, MA, USA). Analysis was guided by Braun and Clarke (2006) framework for thematic analysis, with the two researchers familiarising themselves with the data by re-reading transcripts and developing initial ideas and codes (Braun and Clarke, 2006). Open coding was used to assign the data to codes which were then grouped into categories and, in turn, themes. Themes, categories and codes were conﬁrmed through discussion between the two researchers to maintain conﬁrmability and enhance dependability. The outcome data (means and standard deviations) were calculated using SPSS 22 software. The outcome data set will be available in on Open Science framework (https://osf.io/fbe69/).

## Results

### Feasibility and acceptability of the face-to-face intervention

Table 2 represents a summary of the feasibility, acceptability and outcome data. Recruiting for the study was a simple process and consent was obtained from the required number of participants over a three-week period. The retention rate for the *TG* over the course of the face-to-face intervention was excellent at 100%, and mean attendance was high at 8.2. The retention rate for the *CG*, however, was relatively low at 70%.

**Table 2.** Intervention feasibility, acceptability, and outcome data.

Intervention feasibility data	Outcome
Recruitment time	3 weeks
Attendance rates	8.2 clients per session
Drop-out rates	*TG:* 0% *CG:* 30%
Intervention acceptability data	Focus group quotes
Practical applications	•“I liked the DNA stuff…a good thing to just have at the back of your mind and then it will come to you naturally when you need it” •‘The bracelet was fun to make. When I brought it home I put it on my cabinet and when I looked at it I remembered I was in the group’.
Social and emotional benefits	•Enjoyed meeting new people •Having the opportunity to express your feelings •Trying out different scenarios •Learning to be adventurous
Suggestions for improvement	•More movement activities •Exercises to help understand the model •More games
Intervention outcome data	Summary of outcomes
Comparing TG and CG data at baseline versus 6 weeks	•Minimal change in QoL, mindfulness and diabetes distress scores
Comparing TG only across 3 time points: QoL	•QoL increased over time
Comparing TG only across 3 time points: mindfulness	•Mindfulness scores were stable across all 3 time points
Comparing TG only across 3 time points: diabetes distress	•Diabetes distress lowered at 6 weeks post-intervention, though returned to baseline at 12 weeks

All 10 *TG* participants took part in the focus group, duration 25 minutes When asked to rate the intervention anonymously as to whether it was worthwhile, nine out of 10 participants agreed that it had been worthwhile, while one participant was not sure if they had benefited from the intervention. One participant commented:

Coming here I was a little bit sceptical as to how it was going to work and I felt a bit awkward and a little weird but the whole thing was good, the activities were good and I felt I learnt stuff.

The facilitator and attending psychologist noted that some participants were reluctant to share their reflections in the focus group. Gentle encouragement was used; however, the result was that the collected qualitative data contained a limited number of quotes. Thematic analysis of the focus group data revealed the following themes.

#### Practical applications

Participants commented on benefiting from things they learnt in the groups that they could put into practice in their everyday life. Participants agreed that the bracelet activity, wherein they spelled out one of their values with beads on a bracelet and wore it during the week, was useful as a concrete way to remind themselves of their chosen value and of being in the group when away from it.

The bracelet was fun to make. When I brought it home I put it on my cabinet and when I looked at it I remembered I was in the group…

Another participant liked the activity wherein they identified an action that was associated with their chosen value. A further participant spoke about liking the DNA-V model and the possibility of recalling it when needed:

I liked the DNA stuff…a good thing to just have at the back of your mind and then it will come to you naturally when you need it

#### Social and emotional benefits

Participants commented on several benefits related to their social and emotional wellbeing that they accrued simply from being in a group that acted as a safe place to talk with others who share a commonality. For example, participants said they enjoyed ‘*meeting new people*’, having the opportunity to ‘*express your feelings*’, ‘*try out different scenarios*’, and ‘*learning to be adventurous*’.

#### Suggestions for improvement

Participants suggested incorporating more physical activities and practical exercises to help them understand and think about some of the concepts. It was also suggested that more games should be integrated into the group sessions, and less talking.

### Intervention outcome data

#### TG and CG at baseline and six-weeks

Table 3 represents the outcome data at baseline and 6 weeks, for the *TG* and *CG*. The *TG* QoL scores increased from baseline to 6 weeks, though the *CG* remained the same. The Diabetes Distress scores marginally decreased for the *TG* and *CG*. Mindfulness scores slightly decreased for the *CG*, but remained fairly stable for the *TG*.

**Table 3.** ACT intervention outcome data (*M* and *SD*).

	Baseline (0 weeks)	6 weeks	12 weeks
*Quality of life*			
TG	41.30 (7.39)	42.50 (10.31)	44.40 (7.50)
CG	41.20 (7.25)	40.14 (5.18)	
*Mindfulness*			
TG	25.40 (4.55)	25.50 (3.60)	25.10 (5.76)
CG	26.40 (5.89)	25.43 (5.53)	
*Diabetes distress*			
TG	39.90 (14.33)	37.80 (12.53)	39.60 (15.15)
CG	38.80 (11.95)	35.71 (10.28)	

#### TG only across 3 time points

Table 3 also reports the outcome data across the 3 time points for *TG* only. QoL was higher at 6 weeks post-intervention than at baseline and was highest at 12 weeks post-intervention. Diabetes Distress was lower at 6 weeks post-intervention, than at baseline. At 12 weeks post-intervention, distress levels had returned to baseline. Mindfulness remained stable at all 3 time points.

## Discussion

This pilot and feasibility study aimed to explore the feasibility and acceptability of a face-to-face ACT intervention in adolescents with T1D and pilot methods for a larger RCT.

### Summary of main findings

Recruitment for the intervention was an easy process with the parents of the adolescents we contacted indicating that this type of intervention was needed. At the time, group psychological interventions specifically for adolescents with T1D were not available at the Perth Children’s Hospital. The dropout rate of the control group was of some concern and could be addressed in the future with consideration of an active or waitlist control group. Participants receiving the ACT intervention generally reported being satisfied with the intervention, and this was supported by a high level of attendance at the sessions. Other ACT interventions with adolescents have also reported high levels of acceptability, although not with T1D (Petersen et al., 2023). Participants in our study highlighted social and emotional wellbeing benefits, including meeting other people and learning to be more adventurous in front of others. Participants also liked the practical activities that provided them with something to recall in their daily lives. Participants wanted more movement and games in the sessions and less talking. This would need to be considered in a future intervention. Expression of ACT ideas through movement and practical examples is particularly important for keeping adolescents engaged in sessions (Hayes et al., 2010).

The outcome data suggests that there is a trend towards improvements in QoL for adolescents with T1D. This is in line with findings from a review of ACT interventions for children (Swain et al., 2015). The treatment group also reported improvements in diabetes distress during the intervention period, though it should be noted that this was not statistically tested in this study due to the small sample size. Trends from this study also indicated that improvements in diabetes distress may be lost post intervention. It is possible that adolescents may require booster sessions to sustain any improvements in diabetes distress.

There were no trends indicating any movement in self-reported mindfulness for participants in the face-to-face intervention. A recent review of mindfulness-based interventions for paediatric populations with diabetes concluded that while the evidence is limited, there are sufficient positive signals to suggest the benefit of more rigorous testing of mindfulness programs for this population (Inverso et al., 2022). In our study explicit mindfulness activities represented a small part of each session as it was deemed that these need to be kept short while adolescents are in the beginning stages of learning the skill, however, our data suggest that the chosen mindfulness activities were not adequate to produce meaningful change. Alternatively, the participants may have required additional support from their parents, including further practice of skills at home.

### Alignment with existing research

Few studies have focussed on investigating the feasibility of using ACT with adolescents with T1D. However, it has been found to be an acceptable and highly suitable intervention for adolescents without a chronic condition (Hadlandsmyth et al., 2013; Harris, 2019). The trend towards improvement in diabetes distress in this pilot is supported by literature in adults with diabetes who have participated in an ACT intervention (Maghsoudi et al., 2019; Ngan et al., 2021). Additionally there is evidence that psychological flexibility in adults with T1D may be associated with a decrease in diabetes distress (Nicholas et al., 2022). However, unlike previous studies (Guo et al., 2019), it is possible that these improvements may be lost post-intervention, as trends indicated in this study. It is also possible that diabetes distress acts as a potential covariate for the effectiveness of an ACT intervention, with previous studies suggesting that mindfulness-based interventions are most effective for individuals with high levels of diabetes distress at baseline (Guo et al., 2019; Schmidt et al., 2018).

### Strengths and limitations

A strength of the study was that the facilitator was able to deliver the protocol truly, given the regular attendance of the participants and the manageable size of the group. Another strength was the use of a protocol specifically designed for adolescents. Adult tested protocols, that may be developmentally inappropriate, are often used in ACT interventions for adolescents (Petersen et al., 2024). A key limitation was the small sample size, limiting the generalisability of the results. Additionally, while focus groups can be a valuable tool for encouraging discussion and helping adolescents share their internal views (Akyıldız and Ahmed, 2021), in this case participants were reluctant to speak in the group. It may have been more beneficial to collect individual data through open-ended survey questions. Further, there was no control comparison for the 12-week time-point, which would have provided insight into the long-term impact of the intervention. Additionally, we did not include a measure of psychological flexibility, though a larger scale RCT may look to test the mediation effects of psychological flexibility on treatment outcomes (Wicksell et al., 2010). Another limitation was not knowing to what extent the group effect contributed to positive outcomes.

### Implications for future research

ACT interventions have the potential to improve the psychological and physical health outcomes for individuals with TID (Alho et al., 2022), and provides an important avenue for future research. A larger scale RCT would be feasible, though a few minor modifications would be required. These modifications would include further parental involvement in the intervention and additional outcome measures. Additionally, a future RCT may consider having a waitlist control group, or an active control group, to improve control group dropout rates, and provide a stronger evidence base for the efficacy of the intervention. A greater focus on psychological flexibility and building resilience may be necessary in future interventions as this aligns with a study of people with diabetes (types 1 and 2) who were involved in the READY program (Ryan et al., 2020). It may have been more relevant to measure changes in psychological flexibility, rather than mindfulness, using a scale such as the Diabetes Acceptance and Action Scale for Children and Adolescents (Greco and Hart, 2006).

This study aimed to pilot the feasibility and acceptability of a six-week tailored ACT intervention for adolescents with T1D. Through this study, we determined that that this intervention was largely accepted by study participants. Overall, this study provides evidence to support a larger scale randomised controlled trial to investigate the efficacy of a face-to-face ACT intervention for adolescents with T1D and other chronic illnesses.

## References

[bibr1-13591053251328961] Akyıldız ST Ahmed KH (2021) An overview of qualitative research and focus group discussion. International Journal of Academic Research in Education 7: 1–15.

[bibr2-13591053251328961] Alho I Joro M Juntunen L , et al. (2021) Adolescents with poorly controlled type 1 diabetes: Psychological flexibility is associated with the glycemic control, quality of life and depressive symptoms. Journal of Contextual Behavioral Science 19: 50–56.

[bibr3-13591053251328961] Alho I Lappalainen P Muotka J , et al. (2022) Acceptance and commitment therapy group intervention for adolescents with type 1 diabetes: A randomized controlled trial. Journal of Contextual Behavioral Science 25: 153–161.

[bibr4-13591053251328961] Australian Bureau of Statistics (2018) Socio-economic indexes for areas (SEIFA) 2016. Accessed July 3, 2019. Available at: https://www.abs.gov.au/ausstats/abs@.nsf/mf/2033.0.55.001

[bibr5-13591053251328961] Baer RA (2003) Mindfulness training as a clinical intervention: A conceptual and empirical review. Clinical Psychology: Science and Practice 10: 125–143.

[bibr6-13591053251328961] Barreto M Tran TA Gaynor ST (2019) A single-session of acceptance and commitment therapy for health-related behavior change: An open trial with a nonconcurrent matched comparison group. Journal of Contextual Behavioral Science 13: 17–26.

[bibr7-13591053251328961] Barry-Menkhaus SA Wagner DV Riley AR (2020) Small interventions for big change: Brief strategies for distress and self-management amongst youth with type 1 diabetes. Current Diabetes Reports 20: 3. 32002682 10.1007/s11892-020-1290-7 PMC7083649

[bibr8-13591053251328961] Bluth K Campo RA Pruteanu-Malinici S , et al. (2016) A school-based mindfulness pilot study for ethnically diverse at-risk adolescents. Mindfulness 7: 90–104. 27034729 10.1007/s12671-014-0376-1 PMC4809539

[bibr9-13591053251328961] Boggiss A Consedine N Schache K , et al. (2020) A brief self-compassion intervention for adolescents with type 1 diabetes and disordered eating: A feasibility study. Diabetic Medicine 37: 1854–1860. 32614482 10.1111/dme.14352

[bibr10-13591053251328961] Braun V Clarke V (2006) Using thematic analysis in psychology. Qualitative Research in Psychology 3: 77–101.

[bibr11-13591053251328961] Chantzaras A Yfantopoulos J (2022) Association between medication adherence and health-related quality of life of patients with diabetes. Hormones 21: 691–705. 36219341 10.1007/s42000-022-00400-y PMC9552716

[bibr12-13591053251328961] Chew B Mukhtar F Sherina M , et al. (2015) The reliability and validity of the Malay version 17-item Diabetes Distress Scale. Malaysian Family Physician: The Official Journal of the Academy of Family Physicians of Malaysia 10: 22. 27099658 PMC4826578

[bibr13-13591053251328961] Chin YW Lai PSM Chia YC (2017) The validity and reliability of the English version of the diabetes distress scale for type 2 diabetes patients in Malaysia. BMC Family Practice 18: 1–8. 28219325 10.1186/s12875-017-0601-9 PMC5319150

[bibr14-13591053251328961] Desai AD Zhou C Stanford S , et al. (2014) Validity and responsiveness of the pediatric quality of life inventory (PedsQL) 4.0 generic core scales in the pediatric inpatient setting. JAMA Pediatrics 168: 1114–1121. 25347549 10.1001/jamapediatrics.2014.1600

[bibr15-13591053251328961] De Wit M Delemarre-van de Waal HA Bokma JA , et al. (2008) Monitoring and discussing health-related quality of life in adolescents with type 1 diabetes improve psychosocial well-being: A randomized controlled trial. Diabetes Care 31: 1521–1526. 18509204 10.2337/dc08-0394 PMC2494630

[bibr16-13591053251328961] Doorley JD Goodman FR Kelso KC , et al. (2020) Psychological flexibility: What we know, what we do not know, and what we think we know. Social and Personality Psychology Compass 14: 1–11.

[bibr17-13591053251328961] Eva AL Thayer NM (2017) Learning to BREATHE: A pilot study of a mindfulness-based intervention to support marginalized youth. Journal of Evidence-Based Complementary & Alternative Medicine 22: 580–591. 29228794 10.1177/2156587217696928 PMC5871269

[bibr18-13591053251328961] Farm BAS Perwitasari DA Thobari JA , et al. (2017) Translation, revision, and validation of the diabetes distress scale for Indonesian type 2 diabetic outpatients with various types of complications. Value in Health Regional Issues 12: 63–73. 28648318 10.1016/j.vhri.2017.03.010

[bibr19-13591053251328961] Geist R Grdisa V Otley A (2003) Psychosocial issues in the child with chronic conditions. Best Practice & Research Clinical Gastroenterology 17: 141–152. 12676111 10.1016/s1521-6918(02)00142-7

[bibr20-13591053251328961] Gentili C Rickardsson J Zetterqvist V , et al. (2019) Psychological flexibility as a resilience factor in individuals with chronic pain. Frontiers in Psychology 10: 1–11. 31551871 10.3389/fpsyg.2019.02016 PMC6734029

[bibr21-13591053251328961] Greco L Hart T (2006) Diabetes acceptance and action scale for children and adolescents (DAAS). In: Ciarrochi J Bilich L (eds) Acceptance and Commitment Therapy. Measures Package: Process measures of potential relevance to ACT. New York, NY: Wiley, pp.137–159.

[bibr22-13591053251328961] Greco LA Baer RA Smith GT (2011) Assessing mindfulness in children and adolescents: Development and validation of the child and adolescent mindfulness measure (CAMM). Psychological Assessment 23: 606. 21480722 10.1037/a0022819

[bibr23-13591053251328961] Guo J Wang H Luo J , et al. (2019) Factors influencing the effect of mindfulness-based interventions on diabetes distress: A meta-analysis. BMJ Open Diabetes Research and Care 7: e000757. 10.1136/bmjdrc-2019-000757 PMC6936501 31908794

[bibr24-13591053251328961] Hadlandsmyth K White KS Nesin AE , et al. (2013) Proposing an Acceptance and Commitment Therapy intervention to promote improved diabetes management in adolescents: A treatment conceptualization. International Journal of Behavioral Consultation and Therapy 7: 12.

[bibr25-13591053251328961] Harris E (2019) Acceptance and Commitment Therapy for child and adolescent mental well-being: A systematic review and an acceptability and feasibility study of a universal intervention. PhD Thesis, Cardiff University, Cardiff.

[bibr26-13591053251328961] Hayes LL Bach PA Boyd CP (2010) Psychological treatment for adolescent depression: Perspectives on the past, present, and future. Behaviour Change 27: 1.

[bibr27-13591053251328961] Hayes SC Strosahl KD Bunting K , et al. (2004) What is acceptance and commitment therapy? In: Hayes SC Strosahl KD (eds) A Practical Guide to Acceptance and Commitment Therapy. Boston, MA: Springer, pp.3–29.

[bibr28-13591053251328961] Inverso H Moore HR Lupini F , et al. (2022) Mindfulness-based interventions: Focus on pediatric type 1 and type 2 diabetes. Current Diabetes Reports 22: 493–500. 35984566 10.1007/s11892-022-01492-x PMC10976454

[bibr29-13591053251328961] Kashdan TB Rottenberg J (2010) Psychological flexibility as a fundamental aspect of health. Clinical Psychology Review 30: 865–878. 21151705 10.1016/j.cpr.2010.03.001 PMC2998793

[bibr30-13591053251328961] Kruger R Casey M (2008) Focus Groups: A Practical Guide for Applied Research. Thousand Oaks, CA: Sage.

[bibr31-13591053251328961] Kuby AK McLean N Allen K (2015) Validation of the child and adolescent mindfulness measure (CAMM) with non-clinical adolescents. Mindfulness 6: 1448–1455.

[bibr32-13591053251328961] Loseby P Schache K Cavadino A , et al. (2022) The role of protective psychological factors, self-care behaviors, and HbA1c in young adults with type 1 diabetes. Pediatric Diabetes 23: 380–389. 34967089 10.1111/pedi.13306

[bibr33-13591053251328961] Maghsoudi Z Razavi Z Razavi M , et al. (2019) Efficacy of acceptance and commitment therapy for emotional distress in the elderly with type 2 diabetes: A randomized controlled trial. Diabetes, Metabolic Syndrome and Obesity: Targets and Therapy 12: 2137. 31802921 10.2147/DMSO.S221245 PMC6802537

[bibr34-13591053251328961] Neff KD (2012) The science of self-compassion. Compassion and Wisdom in Psychotherapy 1: 79–92.

[bibr35-13591053251328961] Ngan HY Chong YY Chien WT (2021) Effects of mindfulness-and acceptance-based interventions on diabetes distress and glycaemic level in people with type 2 diabetes: Systematic review and meta-analysis. Diabetic Medicine 38: e14525. 10.1111/dme.14525 33438251

[bibr36-13591053251328961] Nicholas JA Yeap BB Cross D , et al. (2022) Psychological flexibility is associated with less diabetes distress and lower glycated haemoglobin in adults with type 1 diabetes. Internal Medicine Journal 52: 952–958. 33646630 10.1111/imj.15250

[bibr37-13591053251328961] Petersen JM Davis CH Renshaw TL , et al. (2023) School-based acceptance and commitment therapy for adolescents with anxiety: A pilot trial. Cognitive and Behavioral Practice 30: 436–452.

[bibr38-13591053251328961] Petersen JM Ona PZ Twohig MP (2024) A review of acceptance and commitment therapy for adolescents: Developmental and contextual considerations. Cognitive and Behavioral Practice 31: 72–89.

[bibr39-13591053251328961] Pinquart M (2020) Health-related quality of life of young people with and without chronic conditions. Journal of Pediatric Psychology 45: 780–792. 32642762 10.1093/jpepsy/jsaa052

[bibr40-13591053251328961] Polonsky WH Fisher L Earles J , et al. (2005) Assessing psychosocial distress in diabetes: Development of the diabetes distress scale. Diabetes Care 28: 626–631. 15735199 10.2337/diacare.28.3.626

[bibr41-13591053251328961] Prentice K Rees C Finlay-Jones A (2021) Self-compassion, wellbeing, and distress in adolescents and young adults with chronic medical conditions: The mediating role of emotion regulation difficulties. Mindfulness 12: 2241–2252. 34335989 10.1007/s12671-021-01685-7 PMC8311066

[bibr42-13591053251328961] Prevedini AB Presti G Rabitti E , et al. (2011) Acceptance and commitment therapy (ACT): The foundation of the therapeutic model and an overview of its contribution to the treatment of patients with chronic physical diseases. Giornale Italiano di Medicina del Lavoro ed Ergonomia 33: A53–A63. 21488484

[bibr43-13591053251328961] Rausch JR Hood KK Delamater A , et al. (2012) Changes in treatment adherence and glycemic control during the transition to adolescence in type 1 diabetes. Diabetes Care 35: 1219–1224. 22474040 10.2337/dc11-2163 PMC3357213

[bibr44-13591053251328961] Rayner M Hayes LL Ciarrochi J (2017) Write your own DNA: A group program to help young people live with vitality and strength. Accessed March 15, 2019. Available at: https://thrivingadolescent.com/

[bibr45-13591053251328961] Rechenberg K Whittemore R Holland M , et al. (2017) General and diabetes-specific stress in adolescents with type 1 diabetes. Diabetes Research and Clinical Practice 130: 1–8. 28551480 10.1016/j.diabres.2017.05.003 PMC5608607

[bibr46-13591053251328961] Ryan AK Pakenham KI Burton NW (2020) A pilot evaluation of a group acceptance and commitment therapy-informed resilience training program for people with diabetes. Australian Psychologist 55: 196–207.

[bibr47-13591053251328961] Sawyer S Duncan R Drew S (2007) Adolescents with chronic disease: The double whammy. Australian Family Physician 36: 622. 17676185

[bibr48-13591053251328961] Schmidt C van Loon BP Vergouwen A , et al. (2018) Systematic review and meta-analysis of psychological interventions in people with diabetes and elevated diabetes-distress. Diabetic Medicine 35: 1157–1172. 10.1111/dme.13709 29896760

[bibr49-13591053251328961] Shomaker LB Bruggink S Pivarunas B , et al. (2017) Pilot randomized controlled trial of a mindfulness-based group intervention in adolescent girls at risk for type 2 diabetes with depressive symptoms. Complementary Therapies in Medicine 32: 66–74. 28619307 10.1016/j.ctim.2017.04.003 PMC5705100

[bibr50-13591053251328961] Sirois FM Molnar DS Hirsch JK (2015) Self-compassion, stress, and coping in the context of chronic illness. Self and Identity 14: 334–347.

[bibr51-13591053251328961] Swain J Hancock K Dixon A , et al. (2015) Acceptance and Commitment Therapy for children: A systematic review of intervention studies. Journal of Contextual Behavioral Science 4: 73–85.

[bibr52-13591053251328961] van Son J Nyklíček I Nefs G , et al. (2015) The association between mindfulness and emotional distress in adults with diabetes: Could mindfulness serve as a buffer? Results from Diabetes MILES: The Netherlands. Journal of Behavioral Medicine 38: 251–260. 25164478 10.1007/s10865-014-9592-3

[bibr53-13591053251328961] Varni JW Seid M Rode CA (1999) The PedsQL™: Measurement model for the pediatric quality of life inventory. Medical Care 37(2): 126–139. 10024117 10.1097/00005650-199902000-00003

[bibr54-13591053251328961] Vaughn S Schumm JS Sinagub J , et al. (1996) Focus Group Interviews in Education and Psychology. London: Sage.

[bibr55-13591053251328961] Whitehead AL Julious SA Cooper CL , et al. (2016) Estimating the sample size for a pilot randomised trial to minimise the overall trial sample size for the external pilot and main trial for a continuous outcome variable. Statistical Methods in Medical Research 25: 1057–1073. 26092476 10.1177/0962280215588241 PMC4876429

[bibr56-13591053251328961] Wicksell RK Olsson GL Hayes SC (2010) Psychological flexibility as a mediator of improvement in Acceptance and Commitment Therapy for patients with chronic pain following whiplash. European Journal of Pain 14: 1059 (e1051–1059: e1011). 10.1016/j.ejpain.2010.05.001 20538493

[bibr57-13591053251328961] Winkley K Upsher R Stahl D , et al. (2020) Psychological interventions to improve self-management of type 1 and type 2 diabetes: A systematic review. Health Technology Assessment (Winchester, England) 24: 1. 10.3310/hta24280 PMC7336224 32568666

